# The Preparation Process and Hydration Mechanism of Steel Slag-Based Ultra-Fine Tailing Cementitious Filler

**DOI:** 10.3390/gels9020082

**Published:** 2023-01-18

**Authors:** Siqi Zhang, Bo Wu, Yutong Ren, Zeping Wu, Qian Li, Keqing Li, Minggen Zhang, Junhao Yu, Jialu Liu, Wen Ni

**Affiliations:** School of Civil and Resources Engineering, University of Science and Technology Beijing, Beijing 100083, China

**Keywords:** ultra-fine tailing, desulphurised ash, steel slag, hydrated calcium silicate gel, ettringite

## Abstract

Steel slag, desulphurised ash, desulphurised gypsum and ultra-fine iron tailing sand are common industrial solid wastes with low utilisation rates. Herein, industrial solid wastes (steel slag, desulphurised gypsum and desulphurised ash) were used as the main raw materials to prepare a gelling material and ultra-fine tailing was used as an aggregate to prepare a new type of cementing filler for mine filling. The optimal composition of the cementing filler was 75% steel slag, 16.5% desulphurised gypsum, 8.75% desulphurised ash, 1:4 binders and tailing mass ration and 70% concentration. The compressive strength of the 28-day sample reached 1.24 MPa, meeting the mine-filling requirements, while that of the 90-day sample was 3.16 MPa. The microscopic analysis results showed that a small amount of C_3_A reacted with the sulphate in the desulphurised gypsum to form ettringite at the early stage of hydration after the steel slag was activated by the desulphurisation by-products. In addition, C_2_S produced hydrated calcium silicate gel in an alkaline environment. As hydration proceeded, the sulphite in the desulphurised ash was converted to provide sulphate for the later sustained reaction. Under the long-term joint action of alkali and sulphate, the reactive silica–oxygen tetrahedra and alumina–oxygen tetrahedra depolymerised and then polymerised, further promoting the hydration reaction to generate hydrated calcium silicate gel and ettringite. The low-carbon and low-cost filler studied in this paper represents a new methodology for the synergistic utilisation of multiple forms of solid waste.

## 1. Introduction

Mining activities generate a large amount of solid waste (such as tailings and waste rock) that occupies space, causes environmental pollution and is not conducive to sustainable development for mines. In recent years, there has been an abundance of ore resources with a micro-fine particle size. An ore is crushed and ground to a fine size in mineral processing plants to achieve the monomeric dissociation of useful minerals. This often produces a large number of ultra-fine tailings that have a utilisation rate of <20%. The cement filling technology can effectively reduce the surface stockpiles of tailings and mitigate the surface subsidence during underground mining [1].

**Figure 1:**
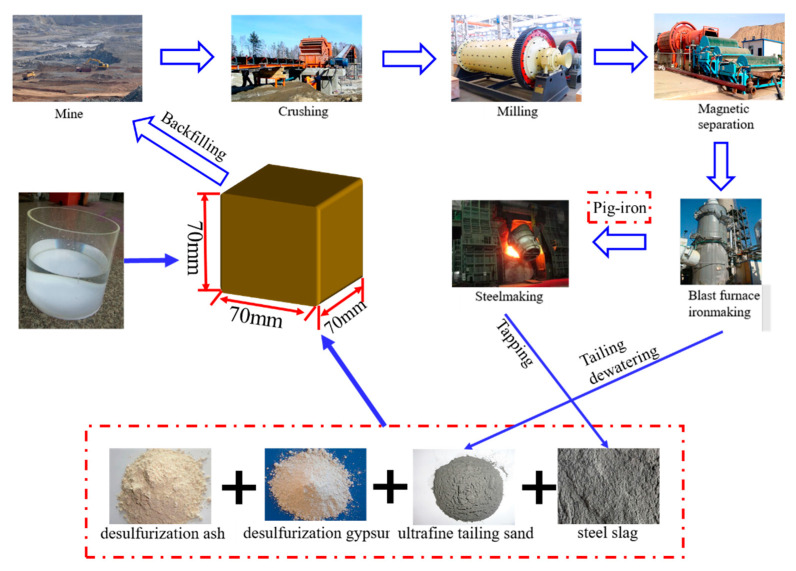
Experimental flow chart.

To maximise iron recovery, a mineral processing plant crushes, grinds and magnetically separates the iron ore through a beneficiation process (Figure 1). This process generates a large amount of tailings, which are then deposited in tailing ponds. Compared with normal tailings, ultra-fine iron tailings have a finer particle size, higher specific surface area and higher potential reactivity. Recently, many scholars have studied the comprehensive treatment of ultra-fine tailings [2]. When ultra-fine tailing sand is used for mine filling, its filling body tends to have lower density, more porosity, lower uniaxial compressive strength and a relatively fluffy structure. This fluffy structure is less abrasive to the pipeline and easily forms a stable, non-separated slurry that considerably reduces pipeline clogging [3]. Cementitious materials (e.g., conventional cement) account for ≥60% of the total cost of cementitious filling. Hence, the search for low-cost, high-quality cementitious materials has become a major research direction.

Some scholars [4,5] found that the use of solid waste as cementitious material can realise similar effects as when using cement. Liu [6] used the alkali NaOH to activate the water-quenching slag used as a cementitious material. They concluded that if the dosage of the new cementitious material is half that of of the cement, with a maintenance age of 28 days, the compressive strength of the new cementitious material filling body in different tailing concentrations is greater than that of the cement filling body. When used as a cementitious material, steel slag can provide an alkaline environment after hydration, helping to improve the compressive strength of a cementitious system [7]. However, steel slag prolongs the setting time of the composite cementitious material, reducing its initial strength [8]. Desulphurised gypsum and desulphurised ash are solid wastes with different compositions produced through flue gas desulphurisation in power plants and steel plants, respectively. Flue gas desulphurised (FGD) gypsum exhibits high purity and a compact crystal structure after hydration. The sulphur compounds in desulphurised gypsum can participate in hydration reactions and provide sulphate ions in the reaction system of cementitious materials. However, this causes uneven strength and poor water resistance, limiting the applications of FGD gypsum [9,10]. In comparison, FGD ash is more difficult to utilise, and only a few studies in China have investigated FGD ash and its applications. Some studies have indicated that cement containing desulphurised ash can be used as a solidifying agent in landfills [11]. Yang et al. [12] studied full tailing sand filling cementitious material as well as desulphurised ash and proposed a key technology for developing a new type of filling cementitious material based on desulphurised ash. Wen et al. [13] reported that the weak acids formed from the hydrolysis of the sulphide in FGD ash slag could neutralise some of the alkaline materials, affording a weaker alkaline environment in the slurry and a decrease in the strength of the cementitious material. Cement blended with FGD ash exhibited superior strength than silicate cement using the modification method of grinding. However, the strength of the blended cement decreased when the FGD ash content exceeded 10% [14]. Our previous study [15] showed that the 28-day compressive strength of the metallurgical slag excited by FGD ash was the same as that excited by FGD gypsum, with the possibility of sulphite converting to sulphate under room-temperature conditions during hydration.

Herein, the synergistic effects of four solid wastes, i.e., ultra-fine tailing sand, steel slag, desulphurised ash and desulphurised gypsum, were investigated. The orthogonal-test method was used to investigate the relation between the strength of the filler and different cementitious material ratios, the amount of tailing sand and admixtures. Based on the experimental results, the optimum ratio is selected to obtain a filler meeting the basic requirements of the mine filler standard. The hydration mechanism of the maintained samples was determined to provide a theoretical basis for multi-solid-waste synergy for the less utilised bulk solid waste.

## 2. Results

### 2.1. Strength and Flow Test

Table 1 shows the results of the strength and flow test via the orthogonal test. All the test blocks exhibited no strength on day 3, and the strength observed was not satisfactory. On day 28, four groups (groups 3, 5, 6 and 7) achieved a strength of 1 MPa, meeting the mine-filling requirements [16]. On day 90, two groups showed increased strengths of 2 and 3 MPa. All the orthogonal test results regarding fluidity were found to be between 172 and 182 mm, meeting the requirements. These orthogonal test results were used to prepare the orthogonal test result analysis table (Table 2). In the table, K1, K2 and K3 denote the sum of the test index values of each factor at three levels. k1, k2 and k3 represent the average compressive strength obtained by dividing K1, K2 and K3 by three for that column, respectively, and R denotes the extreme difference in the factor.

**Figure 2:**
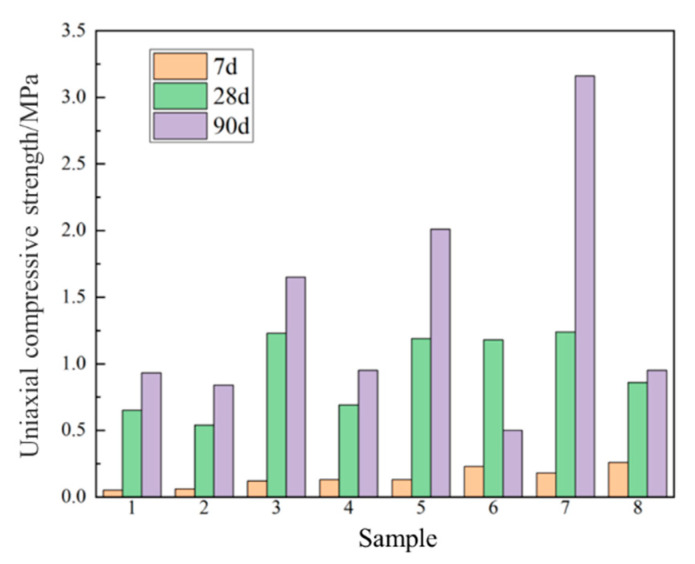
Compressive strength of different samples at different ages.

Based on the magnitude of R values of the three factors, the order of influence of each factor on the strength of the orthogonal test was found as the mass ratio of binders to tailing mass ration > ratio of steel slag to desulphurisation by-products > ratio of desulphurised gypsum: desulphurised ash. Figure 2 is based on the data in Table 1. Group 7 showed optimal long-term strength (day 28: 1.24 MPa and day 90: 3.16 MPa), satisfying the requirement that the 28-day strength should be >1 MPa, and good flow properties. Thus, group 7 was found to be the optimal group for the orthogonal test and subsequent analysis of the hydration mechanism.

### 2.2. XRD Analysis of Sample 7

**Figure 3:**
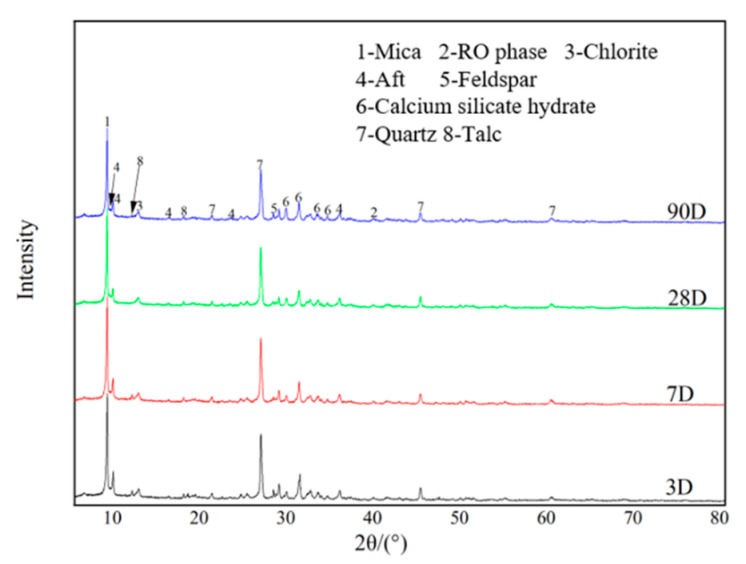
XRD pattern of sample 7 at different curing ages at 20 °C.

Figure 3 shows the XRD patterns of the optimal-ratio sample 7 over 3~90 days. The figure shows that hydrated calcium silicate gel, ettringite and other substances were the main hydration products. By contrast, quartz, feldspar, mica, talc, etc., were the main minerals in the sample. On day 3, spectral peaks of hydrated calcium silicate gel and ettringite could be observed for sample 7, indicating the occurrence of hydration on day 3. However, the formed gel products and ettringite exhibited low content, resulting in the low strength of the sample on day 3. On day 7, quartz and feldspar showed a slight decrease in spectral peaks. The spectral peak of talc observed at 12° disappeared with increasing maintenance age, while those of ettringite and hydrated calcium silicate gel increased. This could be because quartz and talc were involved in the hydration reaction, further developing the gel generated by the reaction of dicalcium silicate, which is closely related to the increase in strength during this period. On day 28 and day 90, the diffraction peaks of quartz further decreased, and hydrated calcium silicate gel was generated under alkaline conditions [17]. New diffraction peaks of ettringite can be observed at 8°–9°, presumably due to the oxidation of subsulphate to form sulphate at the late stage of hydration, which is involved in ettringite formation. At 28~90 days, the spectral peaks of the hydrated calcium silicate gel became denser and the spectral peaks of ettringite increased. In addition, the intensity of the later phase increased compared to the earlier phase. The microscopic level may be due to the denser hydrated calcium silicate gel and wrapping of the ettringite to create a more compact structure [18].

### 2.3. Fourier-Transformed Infrared Spectroscopy Analysis of Sample 7

**Figure 4:**
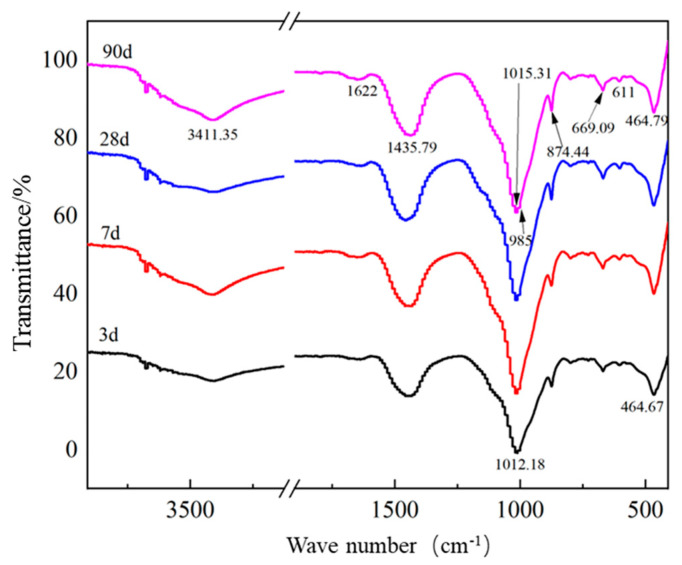
FT-IR spectra of sample 7 at different ages cured at 20 °C.

Figure 4 shows the FT-IR spectra of sample 7 at different hydration ages. The absorption bands located at 3411.35 and 1622 cm^−1^ correspond to the stretching and bending vibrations of the O–H bond in crystalline water, respectively [19]. As shown by the spectral peaks, the two absorption bands broadened while the transmittance decreased with time. This observation confirms the continuous formation of ettringite with the hydrated calcium silicate gel as the hydration process advanced, as observed via XRD analysis. The absorption band observed at 669.09 cm^−1^ is associated with the Si–O–Al bond [20] and its transmission decreased at 7, 28 and 90 days. This indicates the separation of SiO_4_ and AlO_4_ tetrahedra from the amorphous oxide [17]. As shown in the IR analysis spectrum, the main absorption bands in the Si-O vibrational band ranged from 900 to 1200 cm^−1^ [21]. As shown in Figure 4, the absorption band observed at 1012.18 cm^−1^ corresponds to the asymmetric stretching vibration of the Si-O bond and shifts toward higher wavenumbers with increasing hydration age. This finding indicates the increased polymerisation of the hydrated silicates and the production of large amounts of hydrated calcium silicate gel in the middle and late stages of hydration [21,22]. The vibrational band observed at 611 cm^−1^ represents SO_4_^2−^ [15] generated from the ettringite produced by hydration as well as from unreacted gypsum. The symmetric stretching vibration of SO_3_^2−^ corresponds to the absorption band at 985 cm^−1^, whose permeability increases as the hydration proceeds. The absorption band observed at 464.79 cm^−1^ corresponds to a specific ion SO^2−^ that appeared during the conversion of SO_3_^2−^ into SO_4_^2−^ [15]. This finding suggests SO_3_^2−^ converted into SO_4_^2−^ with time during the hydration process.

### 2.4. SEM-EDS Analysis of Sample 7

**Figure 5:**
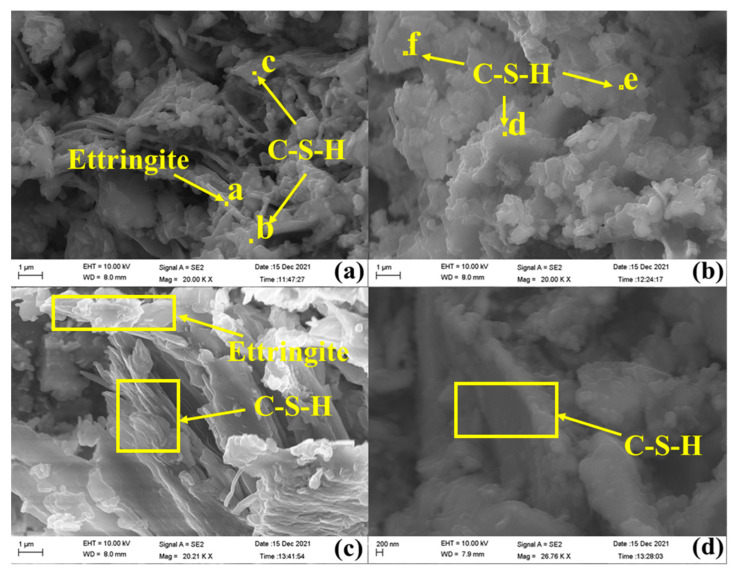
SEM images of sample 7 (**a**,**b**) on day 28 and (**c**,**d**) on day 90.

The SEM results of sample 7 on day 28 and day 90 are shown in Figure 5a–d, respectively. As shown in Figure 5a,b, the gelled system developed on day 28, showing a lamellar shape, and the gel wrapped with ettringite provided a stable structure. As shown in Figure 5c,d, the gel further developed into a lamellar structure with increased volume and stronger wrapping. Ettringite developed into a slender columnar structure with more quantity than on day 28. In addition, ettringite exhibited a thicker and stronger columnar structure on day 90 and was mutually glued with the gel to make the structure denser, accounting for a further increase in the strength.

The results of energy spectrum analysis for each point in Figure 5 are shown in Table 3. Figure 5a shows a long columnar structure of the hydration product that was proved to be chalcocite through EDS analysis. Points b and c marked in Figure 5a represent the gelled material wrapped with ettringite that was found to be hydrated calcium silicate gel using EDS analysis and its calcium–silica ratio at these points was 0.55 and 0.31, respectively. 

### 2.5. TG–DSC Analysis of Sample 7

**Figure 6:**
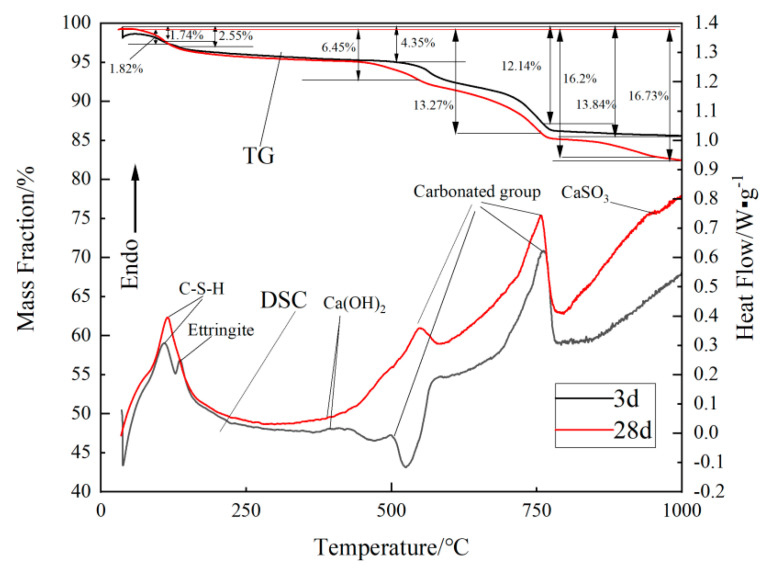
TG–DSC curve of sample 7 obtained at different curing ages at 20 °C.

The TG–DSC curves of sample 7 obtained on day 3 and 28 are shown in Figure 6. On day 3 of hydration, the heat absorption peaks were observed at ≤200 °C. The hydrated calcium silicate gel and ettringite exhibited heat absorption peaks at 109.8 °C and 135.3 °C, respectively [23,24,25,26], and their corresponding TG curves showed mass losses of 1.74% and 2.55%, respectively. After 28 days of hydration, the heat absorption peak of the hydrated calcium silicate gel considerably increased to 114.5 °C, and the mass loss changed to 1.82%. This result indicates that the system produced a large amount of hydrated calcium silicate gel with increasing hydration age. The dehydration temperature of Ca(OH)_2_ was observed as 396.8 °C, proving that the steel slag provided an alkaline environment for the system. The dehydration temperature of pure calcite was ~750 °C, and the peak observed at ~750 °C was due to the decomposition of calcite (CaCO_3_) [27]. The decomposition of CaSO_3_ in the desulphurised ash afforded a small peak corresponding to the DSC curve of the 28-day sample at 950 °C [28,29,30]. In the TG curve, the total mass loss of the sample for 28 days was 16.93% and for 3 days was 13.84%. These results indicate an overall increase in the amount of hydrolysis products during the hydration reaction, which is consistent with the results obtained via SEM, XRD and FT-IR analyses.

### 2.6. XPS Analysis of Sample 7

The changes in excitation energy of the outer second layer of O, S, Al and Ca at different hydration ages of sample 7 were determined via XPS. In addition, the formation and decomposition processes of old and new combinations among the elements during the hydration process were analysed from a microscopic perspective.

#### 2.6.1. Binding Energy of O

**Figure 7:**
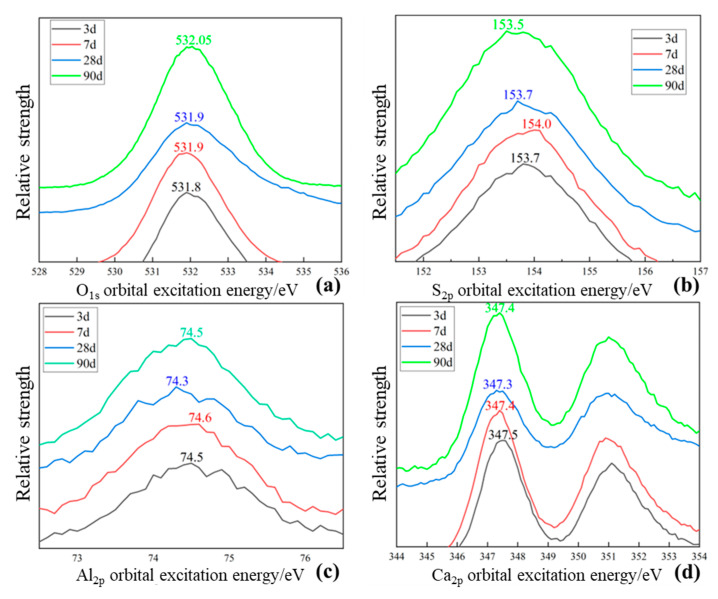
XPS spectra of main elements at different ages.

As shown in Figure 7a, the 1 s orbital excitation energy of O gradually increased from 531.8 eV (on day 3) to 532.05 eV (on day 90) with increasing hydration age. The O_1s_ orbital excitation energy of the non-bridging oxygen bond was 530–530.5 eV, that of the bridging oxygen bond was 531.5–532.7 eV, that of hydroxide was 533–533.5 eV and that of crystalline water molecules was 534 eV [31]. The O_1s_ orbital excitation energy peaks observed between 531.5 and 532.7 eV increased with an increase in hydration age. The low hydration activity in the system caused a small increase in the excitation energy from 3 to 7 days. From 7 to 28 days, the peak broadened owing to the generation of gel-like hydration products and may represent disordered silicate structures [32]. The increase in the hydration age from 28 to 90 days indicates the existence of hydration reactions in the active minerals from the tailing sand in the late stage of hydration. Thus, some of the non-bridging oxygen bonds of the gelling material transformed into bridging oxygen bonds during hydration, and the polymerisation of the hydrated silicate increased. This considerably reduced the porosity and improved the strength of the gel products. 

#### 2.6.2. Binding Energy of S

As shown in Figure 7b, the S_2p_ orbital excitation energy initially increased and then decreased. Before the hydration reaction, S existed in gypsum dihydrate crystals and gypsum hemihydrate crystals in the form of sulphate and sulphite. During hydration, four oxygen atoms formed covalent bonds around S atoms, two water molecules formed hydrogen bonds with the O atoms in sulphate and calcium atoms formed coordination bonds with S. In addition, the desulphurised ash contained sulphite, because of which the binding energy of S was found to be low initially. The S_2p_ orbital excitation energy increased at the beginning of hydration because of the oxidation of sulphite. With the progress in hydration, the oxidation of sulphite in the desulphurised ash provided a portion of sulphate for the system. During the hydration reaction, S was continuously transferred to the hydration product, ettringite crystals, in the form of sulphate distributed in {Ca_6_[Al(OH)_6_]_2_·24H_2_O}^6+^ crystal columns. Ettringite contained a large amount of crystalline water and Al^3+^ and Ca^2+^, and the metal cations contributed electron clouds to S through O, increasing the density of the out-of-nuclear electron cloud of S, decreasing the excitation energy of S_2p3/2_ orbitals. 

#### 2.6.3. Binding Energy of Al 

As shown in Figure 7c, the peak shape of Al_2p_ orbital excitation energy was wider compared with that of other elements. The Al_2p_ orbital excitation energy increased from 74.4 to 74.6 eV during 3–7 days. After 28 days, the excitation energy decreased to 74.3 eV, probably because of the small total mass fraction of Al in the raw material and a lower amount of Al entered the reaction system, which might result in low excitation energy. 

#### 2.6.4. Binding Energy of Ca 

As shown in Figure 7d, the Ca_2p_ orbital excitation energy exhibited two characteristic peaks, belonging to the Ca_2p3/2_ (left) and Ca_2p5/2_ orbitals (right). The Ca_2p_ orbital excitation energy gradually decreased with increasing hydration age, indicating that hydration generated more non-metallic ions or electron-withdrawing groups around Ca. The increase in excitation energy of Ca from 28 to 90 days of hydration might be related to the carbonisation of hydration products, where Ca could be transferred to calcium carbonate to reduce the number of surrounding coordination bonds and enhance its ionicity.

### 2.7. Discussion

The increase in strength of the samples is mainly attributed to the cementation interactions of various hydration products, such as the C–S–H gel and ettringite, in the cementing system [4,33,34]. C_2_S and C_3_S in the steel slag generated C–S–H and Ca(OH)_2_ during hydration (Equations (1) and (2)), and Ca(OH)_2_ created an alkaline environment for the cementation system, providing OH^−^ and acting as an alkaline excitation source. The oxidation of the sulphite in gypsum generated more stable sulphates, releasing sulphate ions to participate in the hydration reaction. Ca(OH)_2_ and the dissolved SiO_2_ and Al_3_O_2_ reacted with volcanic ash, accompanied by the generation of hydrated calcium silicate and hydrated calcium silicon–aluminate in the process [4,34]. Together with the sulphate excitation of gypsum, it provided more SO_4_^2−^ as well as Ca^2+^ to the system, which continuously reacted with SiO_4_^2−^ and AlO_4_^5−^ in the system to generate ettringite. The voids between ettringite were filled by increasing the amount of hydrated calcium silicate gels, and various hydration products stuck together to increase strength. Gel-like substances, such as hydrated calcium silicate, acted as binders.

C_3_S + H_2_O = C–S–H + Ca(OH)_2_ (1)

C_2_S + H_2_O = C–S–H + Ca(OH)_2_ (2)

Ca(OH)_2_ + H_2_O + SiO_2_ = C–S–H (3)

Ca(OH)_2_ + (n − 1)H_2_O + Al_3_O_2_ + SiO_2_ = CaO• SiO_2_• Al_2_O_3_• nH_2_O (C–A–S–H) (4)

3Ca(OH)_2_ + Al_2_O_3_ + 3(CaSO_4_·2H_2_O) + 23H_2_O = 3CaO·Al_2_O_3_·3CaSO_4_·32H_2_O (Ettringite) (5)

As shown in Figure 3, a small amount of ettringite was formed via hydration after 3 days of curing, indicating that the C_3_A in the steel slag exhibited good hydration activity. In the presence of gypsum, the hydration product C_3_AH_6_ of C_3_A in the steel slag reacted with the sulphate in gypsum to form ettringite [17]. However, the compressive strength of the sample was low owing to the low content of aluminium in steel slag. After 7 days of curing, the ettringite crystals were well developed and could be observed in the XRD pattern (Figure 3). The initial strength was not satisfactory (≤1 MPa) owing to the insignificant role of desulphurised ash in the early stage of hydration and the low content of C_3_A in the steel slag. During the hydration process, C_2_S generated hydrated calcium silicate gel and Ca(OH)_2_, but the content of hydrated calcium silicate gel formed was low in the early stage due to the low content of C_2_S.

After 28 days of curing, ettringite crystals and hydrated calcium silicate gel were further developed [35,36]. The tailing sand was stimulated by the Ca(OH)_2_ formed by the hydration of calcium oxide in the steel slag and started to dissociate the silicate and AlO_4_ tetrahedra. The breakage of CaO bonds in the tailing sand produced more Ca(OH)_2_. Under alkaline conditions, the silicate tetrahedra decomposed into the hydrated calcium silicate gel, releasing the AlO_4_^5−^ tetrahedra linked to the silicate tetrahedra. The hydrated calcium silicate gel was formed owing to the reaction between the dissociated AlO_4_^5−^ tetrahedra and Ca(OH)_2_. At the same time, Ca(OH)_2_ could promote the breaking of the Si–O–Al and O–Si–O bands. The dissolved alumina tetrahedra could react with SO_4_^2−^ and Ca^2+^ ions to form ettringite, and the dissolved silica tetrahedra could participate in the generation of the hydrated calcium silicate hydrate gel [37]. The tetrahedra further reacted with sulphate to form ettringite, because of which the strength of the samples reached 1.24 MPa after 28 days of curing (as shown in Table 1). 

Further extension of the curing time by 90 days increased the amount of hydration products and the polymerization degree. Further, the silicate structure of hydrated calcium silicate gel gradually changed from lamellar to reticulate [33]. Figure 7a shows that the binding energy maxima of O_1s_, S_2p_, Al_2p_ and Ca_2p_ are 531.9, 153.7, 74.3 and 347.3 eV, respectively. The width of the absorption band increases from 1620 cm^−1^ to 3403.31 cm^−1^ (as shown in Figure 6), indicating an increase in the content of crystalline water in the hydration products. The hydrated calcium silicate gel grew on the framework formed by ettringite and wrapped around the ettringite crystals, further filling the pores between the ettringite crystals [36]. Finally, the ettringite crystals crossed in the cemented system, and the hydrated calcium silicate gel became the cementing agent between the ettringite crystals. The XRD pattern (Figure 3) showed that the FGD gypsum completely reacted after 28 days but, owing to the gradual oxidation of SO_3_^2−^ in the FGD ash, the system still contained a large amount of SO_4_^2−^. The results of XPS analysis of S_2p_ (Figure 7b) show that a large amount of ettringite was formed in the system and its intensity increased to 3.16 MPa on day 90 compared with that on day 28 (Table 1). 

## 3. Conclusions

Industrial solid wastes (including steel slag, desulphurised gypsum, desulphurised ash and ultra-fine tailing) were used to develop a new filler with a 28-day strength of 1.24 MPa. We believe that this study can achieve a reduction in industrial solid waste and improve resource utilisation. According to the experimental research, the following conclusions can be drawn:

1. The composition of filling material proportion was 75% steel slag, 16.5% FGD gypsum, 8.5% FGD ash, 1:4 binders and tailing mass ration and 70% concentration. The compressive strength of the samples with this composition was 1.24 MPa on day 28 of hydration and 3.16 MPa on day 90.
2. The complex structure formed by ettringite and hydrated calcium silicate gel mainly contributed to its strength. In the early stage of hydration, a small amount of the hydrated calcium silicate gel was obtained through C_2_S hydration in the slag. The reaction of C_3_A with the SO_4_^2−^ of desulphurised gypsum under an alkaline environment formed needle-like ettringite crystals that developed continuously; however, there was low aluminium content in the slag. As hydration progressed, the surface of ultra-fine iron tailing sand began to dissociate the silicate and AlO_4_^5−^ tetrahedra, and the SO_3_^2−^ in desulphurised ash was converted to SO_4_^2−^ that participates in the hydration reaction. This resulted in the formation of more ettringite with the hydrated calcium silicate gel that had a more complex silicate structure. The hydrated calcium silicate gel adhered to the ettringite crystals and further filled the pores.
3. The effect of desulphurised ash was mainly manifested after 28 days of hydration. With the progress in the reaction, the sulphite ions in the desulphurised ash were converted to generate more sulphate ions, which were the main source of sulphate ions in ettringite at the late stage of hydration. Thus, the addition of desulphurised ash to the system solved the problem of insufficient sulphate content in the late stage and could improve the strength of the sample to a certain extent at this stage.
4. Herein, an effective synergistic method is proposed for utilising difficult-to-use solid wastes, such as ultra-fine tailings, steel slag and desulphurisation by-products. The cost of the prepared mine cemented filler is low, providing new ideas for secondary resource utilisation of solid wastes and green mines.
5. The utilisation of ultra-fine tailing sand needs to be explored, especially in solid-waste-based cementitious systems with high potential for ultra-fine tailing sand applications. Herein, the quantitative analysis of reaction products was less and the investigation of other properties of the samples, such as their curing properties against heavy metals, was missing; the exploration of these aspects will be improved in a future study.

## 4. Materials and Method

### 4.1. Materials

Herein, steel slag, desulphurised gypsum, desulphurised ash and ultra-fine iron tailing sand were used as raw materials. Their chemical compositions are shown in Table 4. The steel slag is an alkaline oxygen slag with a specific surface area of 400(±20) m^2^·kg^−1^. Its alkalinity coefficient [38] ((CaO% + MgO% + Al_2_O_3_%)/(SiO_2_% + Fe_2_O_3_% + MnO%)) is 1.17. Its activity coefficient [39] (quality factor) ((CaO% + MgO% + Al_2_O_3_%)/(SiO_2_% + MnO% + TiO_2_%)) is 5.10. Further, the steel slag also has high activity. The specific surface areas of desulphurised gypsum, desulphurised ash and iron tailing sand are 350(±20), 695 and 590 m^2^ kg^−1^, respectively. The particle size of fine and coarse aggregates is in a range of 2.84~19.9 μm and 19.9~151 μm, respectively.

**Figure 8:**
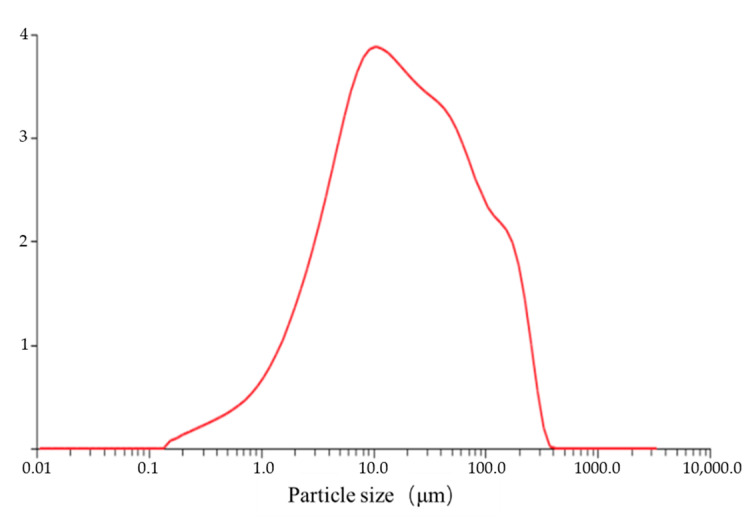
Particle-size distribution of ultra-fine tailing.

The particle-size distribution of ultra-fine iron tailing sand was examined using a laser particle size analyser. Figure 8 shows the particle-size distribution of tailing sand between 4 and 120 μm, in which D_10_ = 2.5 μm, D_50_ = 17 μm and D_90_ = 122 μm. The particle-size classification of tailings is based on the common classification standards of domestic mines (Table 5). Approximately 55% of tailing sand particles have a particle size ≤19 μm, while about 18% particles have a size of ≥74 μm. Therefore, the tailing sand has fine particles that exhibit good water-retention performance. In addition, the uniformity and curvature coefficients are used to evaluate the gradation excellence of the tailing sand. The uniformity factor $CU=\frac{D60}{D10}=12.86$ indicates that the tailing sand has a wide range of particle-size distributions, and the tailing sand particles are uniform and can be easily compacted. The curvature factor is given by $Cc=\frac{D_{30}^{2}}{D10\cdot D60}=1.00$, suggesting that the tailing sand has good continuity.

### 4.2. X-ray Diffraction Analysis of Raw Materials

**Figure 9:**
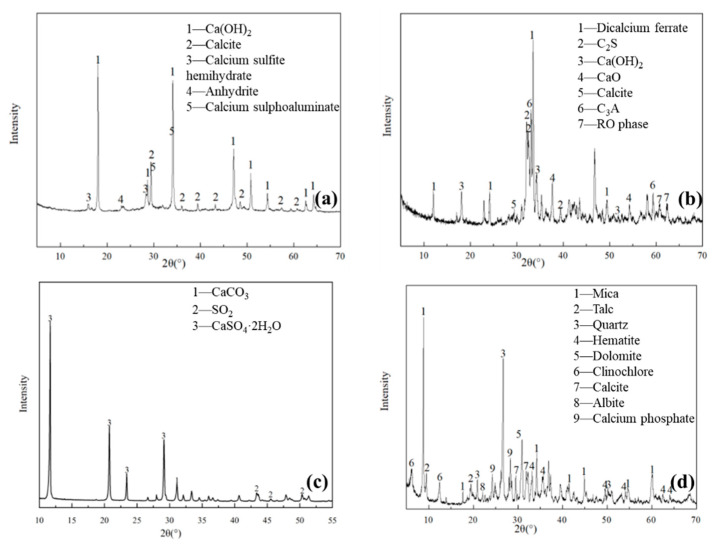
XRD spectra of raw materials: (**a**) desulphurised ash, (**b**) steel slag, (**c**) desulphurised gypsum and (**d**) tailings.

As shown in Figure 9a, the mineral composition of desulphurised ash was dominated by calcium hydroxide, calcite, calcium sulphite hemihydrate and small amounts of hard gypsum and calcium sulphoaluminate. Steel slag mainly contained CaO, Fe_2_O_3_, SiO_2_, MgO and Al_2_O_3_. As shown in the X-ray diffraction (XRD) pattern of steel slag (Figure 9b), the mineral composition included dicalcium ferrate, calcium hydroxide, calcite, dicalcium silicate (C_2_S), calcium oxide, tricalcium aluminate (C_3_A) and the RO phase. As shown in the XRD pattern of desulphurised gypsum (Figure 9c), the mineral composition mainly comprises calcium sulphate dihydrate (CaSO_4_–2H_2_O), which also contained small amounts of CaCO_3_ and SO_2_. Ultra-fine iron tailings mainly comprised SiO_2,_ Fe_2_O_3_ and MgO, followed by CaO and Al_2_O_3_ (Table 5). The sulphur content in the tailings was low and was not majorly involved in the reaction during the filling process. As shown in Figure 9d, the mineral composition of ultra-fine iron tailings was dominated by quartz, mica, talc, dolomite, etc.

### 4.3. Methods

The experiments were conducted at room temperature (20 °C) and the slurry concentration was 70%. Various properties of the paste were affected by the binders/tailing mass ratios, the FGD gypsum/FGD ash ratio and the steel slag/FGD by-product ratio. The levels of each factor are shown in Table 6.

The efficacy coefficient method can effectively evaluate the combination of indicators in the multiple-indicator orthogonal test. If factor i has the best effect, the efficacy coefficient of factor i is 1 (di = 1), and the efficacy coefficients of other factors are the ratio of their effects with respect to the best effect. The total effectiveness coefficient is the arithmetic mean of all factors.

As shown in Table 6, the raw materials satisfying the corresponding conditions were weighed, the appropriate amount of water required to satisfy the slurry concentration of 70% was weighed and then the raw materials were placed into a clean stirring pot and manually stirred for 90 s with slow stirring and 90 s with fast stirring. This was followed by vibration of the mixture, after which the flow was measured according to the national standard GB/T2419-2005 and placed into an appropriate mould to create a block of 70 mm × 70 mm. The block was attached to cling film and cured in a curing chamber at 90% relative humidity and a specific temperature of 20 °C until the test age. The concrete samples were tested for compressive strength at the ages of 3, 7, 28 and 90 days, according to the national standard GB/T50081-2002. The samples were then crushed and analysed at 3, 7, 28 and 90 days of age.

A Japanese Neo Confucianism Ultima IV X-ray diffractometer, with Cu kα radiation, 40 kV voltage and 10 mA current, was used for XRD analysis in the 5° < 2θ < 80° range and at a scanning speed of 8°/min with a step of 0.02°. Fourier-transform infrared (FT-IR) spectroscopy analysis was conducted using a US NICOLET470 Fourier infrared spectrometer, with a wavenumber range of 350~4000 cm^–1^ and a sensitivity of 4 cm^−1^. A thermogravimetry (TG) differential scanning calorimetry (DSC) analyser (NetzschSTA 449C, Germany) was used to measure the mass loss of the sample powder. For this, the temperature was increased from 20 °C to 1010 °C at a rate of 10 °C/min and under N_2_ atmosphere. Scanning electron microscopy (SEM) energy dispersive X-ray spectroscopy (EDS) analysis was conducted using the Key Laboratory of Orogenic Belts and Crustal Evolution, Ministry of Education. SEM observation was conducted on the Suppatm 55 scanning electron microscope produced by the Zeiss Company of Germany and is equipped with a synergy-type spectrometer. The working voltage was 10 kV. X-ray photoelectron spectroscopy (XPS) was used to analyse the chemical state of different elements in the solidified sample and infer the reaction trend of hydration products.

## Figures and Tables

**Table 1:** Orthogonal test results.

Group	Factor			Compressive Strength (MPa)				Fluidity (mm)	d_1_	d_2_	d_3_	d_4_	D
	A	B	C	3d	7d	28d	90d						
1	1	1	1	--	0.05	0.65	0.93	180.5	0.19	0.52	0.29	1.00	0.41
2	1	2	2		0.06	0.54	0.84	178.5	0.23	0.43	0.26	0.98	0.40
3	1	3	3		--	--	--	180.2	--	--	--	0.99	--
4	2	1	2		0.12	1.23	1.65	176.0	0.46	0.99	0.52	0.97	0.69
5	2	2	3		0.13	0.69	0.95	176.5	0.5	0.56	0.30	0.98	0.53
6	2	3	1		0.13	1.19	2.01	180.5	0.5	0.96	0.64	1.00	0.74
7	3	1	3		0.23	1.18	0.50	176.5	0.88	0.95	0.16	0.98	0.60
8	3	2	1		0.18	1.24	3.16	180.5	0.69	1.00	1.00	1.00	0.91
9	3	3	2		0.26	0.86	0.95	172.5	1.00	0.69	0.30	0.96	0.66

A = Steel slag: Desulphurisation by-products; B = FGD gypsum: FGD ash; C = Binders/tailings mass ration; d1 refers to the factor of compressive strength on 7 d in the efficacy coefficient method; d2 refers to the factor of compressive strength on 28 d in the efficacy coefficient method; d3 refers to the factor of compressive strength on 90 d in the efficacy coefficient method; d4 refers to the factor of fluidity in the efficacy coefficient method.

**Table 2:** Analysis of orthogonal test results.

Level	Factor		
	A	B	C
K1	1.77	3.08	6.1
K2	4.61	4.95	3.44
K3	4.61	2.96	1.45
k1	0.590	1.027	2.033
k2	1.537	1.650	1.147
k3	1.537	0.987	0.483
R	0.947	0.663	1.550

The factor of Ki= the sum of compressive strength on 28 d in Table 1 which the number of steel slag to desulfurisation by-product =I; ki = Ki/3; R is the range of k.

**Table 3:** EDS results of sample 7 on day 28 and day 90.

Weight/% Number	O	Mg	Al	Si	S	K	Ca	Fe
a	23.11	6.41	4.15	15.50	9.95	2.27	32.19	6.42
b	39.20	9.53	4.84	17.02	1.20	1.91	9.40	16.90
c	43.38	13.27	2.46	21.80	1.06	—	6.73	11.29
d	46.27	14.86	7.65	20.44	—	6.10	4.68	—
e	37.31	7.68	7.53	20.36	0.66	0.70	6.51	19.25
f	34.22	8.37	6.29	13.92	3.02	—	15.71	18.46

The data presented in the table correspond to the points marked in Figure 5.

**Table 4:** Chemical compositions of raw materials (mass fraction/%).

Material	CaO	SiO_2_	Fe_2_O_3_	MgO	Al_2_O_3_	SO_3_	TiO_2_	K_2_O	Na_2_O	P_2_O_5_	MnO
Steel slag	45.56	10.81	26.26	5.19	2.66	0.55	1.52	0.09	0.05	2.28	1.71
FGD gypsum	31.94	2.36	0.54	0.96	0.49	45.00	0.03	0.11	0.23	0.02	----
FGD ash	56.30	1.02	0.42	0.35	0.38	15.70	0.03	0.13	0.06	0.02	----
Ultra-fine tailing	9.58	34.85	15.88	12.96	7.52	0.26	0.42	2.62	0.61	2.58	0.16

**Table 5:** Common classification standards of tailings in domestic mines.

Classification	Thick		Medium		Thin	
Particle size (μm)	+74	−19	+74	−19	+74	−19
Proportion (%)	>40	<20	20–40	20–55	<20	>50

−19 refers to tailing particles with a particle size of <19 μm; +74 refers to tailing particles with a particle size of >74 μm.

**Table 6:** Orthogonal test of factors and levels.

Level	Factor		
	A	B	C
1	1:1	8:2	1:4
2	2:1	6.5:3.5	1:6
3	3:1	5:5	1:8

## Data Availability

The data presented in this study are available from the corresponding author upon request.

## Abbreviations

DSC	Differential scanning calorimetry
EDS	Energy dispersive X-ray spectroscopy
FGD	Flue gas desulphurisation
SEM	Scanning electron microscopy
XPS	X-ray photoelectron spectroscopy
XRD	X-ray diffraction
